# The Liverpool Care Pathway: a systematic review discarded in cancer patients but good enough for dying nursing home patients?

**DOI:** 10.1186/s12910-017-0205-x

**Published:** 2017-08-09

**Authors:** Bettina S. Husebø, Elisabeth Flo, Knut Engedal

**Affiliations:** 1 Centre for Elderly and Nursing Home Medicine, Department of Global Public Health and Primary Care, University of Bergen, Bergen, Norway; 2 Bergen Municipality, Bergen, Norway; 3 Department of Clinical Psychology, University of Bergen, Bergen, Norway; 4 Norwegian National Advisory Unit on Ageing and Health (Ageing and Health), Vestfold hospital and Oslo universitet hospital, Ullevaal, Oslo, Norway

**Keywords:** Nursing Home, Liverpool Care Pathway, Dementia, Geriatric, Decision-making, End-of-Life-care, Reliability, Validity, Responsiveness

## Abstract

**Background:**

The Liverpool Care Pathway (LCP) is an interdisciplinary protocol, aiming to ensure that dying patients receive dignified and individualized treatment and care at the end-of-life. LCP was originally developed in 1997 in the United Kingdom from a model of cancer care successfully established in hospices. It has since been introduced in many countries, including Norway. The method was withdrawn in the UK in 2013. This review investigates whether LCP has been adapted and validated for use in nursing homes and for dying people with dementia.

**Methods:**

This systematic review is based on a systematic literature search of MEDLINE, CINAHL, EMBASE, and Web of Science.

**Results:**

The search identified 12 studies, but none describing an evidence-based adaption of LCP to nursing home patients and people with dementia. No studies described the LCP implementation procedure, including strategies for discontinuation of medications, procedures for nutrition and hydration, or the testing of such procedures in nursing homes. No effect studies addressing the assessment and treatment of pain and symptoms that include dying nursing home patients and people with dementia are available.

**Conclusion:**

LCP has not been adapted to nursing home patients and people with dementia. Current evidence, i.e. studies investigating the validity and reliability in clinically relevant settings, is too limited for the LCP procedure to be recommended for the population at hand. There is a need to develop good practice in palliative medicine, Advance Care Planning, and disease-specific recommendations for people with dementia.

**Electronic supplementary material:**

The online version of this article (doi:10.1186/s12910-017-0205-x) contains supplementary material, which is available to authorized users.

## Backgrounds

The Liverpool Care Pathway (LCP) is an interdisciplinary procedure developed to make sure that dying patients face sound ethical decision-making regarding treatment and care, meeting their individual physical, psychosocial, and existential needs in their last days of life [1]. Cost-effective treatment, prevention of unnecessary emergency interventions, and hospitalization has been described as important secondary goals [2].

The LCP has been designed to establish vital care interventions for patients and their families in the last days of life and early grieving process [3]. The LCP was developed in the UK nearly 20 years ago, supported by the Royal Liverpool University Trust and Marie Curie Centre Liverpool [1]. The method was intended for use among cancer patients and presumed an open, timely communication between the treating physician, nursing staff, patient and relatives. Appropriate use of LCP requires that the responsible physician makes an accurate assessment of their patient as truly dying. In recent years, the procedure has been adopted in the care of other patient groups than cancer patients, such as chronic kidney disease [4] and burn victims [5]. LCP received broad support, particularly in the UK and is used in 17 other countries, including Norway.

In the UK, “The Gold Standards Framework for Care Homes” (GSFCH) was developed in parallel with the implementation of LCP. The GSFCH offers educational modules, which include preparing communication (Advance Care Planning [ACP]) concerning the appointment of a legal guardian, making antecedent decisions against cardiopulmonary resuscitation, and giving information about the LCP [6]. The initiation and follow-up of ACP communication between patients, relatives and health professionals, including a physician, has been established as a prerequisite for a dignified death in nursing homes (NH) [7, 8].

In England, in 2009, concerns were raised that the use of LCP could possibly contribute to the shortening of some patients’ lives (Delvin K. In: The Daily Telegraph. 2009). It was highlighted that especially old people and patients with other diagnoses than cancer, were put “on the pathway” without adequate medical assessment and without adequate communication to relatives [9, 10]. Concerns were addressed in the media and public debates. The media storm eventually led to the independent review commissioned by the UK Government “More Care, Less Pathway”, the Neuberger report [11]. The report concluded that LCP could in some instances be a suitable procedure, yet, based on unclear implementation strategies and lack of competence, there was a real danger of misjudgements with fatal consequences. In contrast to the UK, an open critical debate never took place in Norway or other countries in Scandinavia. Furthermore, this process had no effect on the use in NHs. Since 2006, LCP has been introduced for use in NHs and among people with dementia [12, 13], and has also been implemented in approximately 270 institutions in Norway. To ensure proper assessment and treatment of pain and distressing symptoms in the dying old, one has to investigate the evidence base for the use of LCP in these settings.

This systematic review aims to investigate the evidence base for the use of LCP in NHs and among dying people with dementia. The review includes studies where the procedure has been developed and adapted for the specific use in this population. More specifically, we aim to investigate the following research questions:

1. Have the LCP been validated and tested for use in dying NH patients and people with dementia?
2. What study designs and methods were employed?
3. Which LCP implementation strategies were used and how were they described?
4. What were the main outcomes of LCP interventions in NHs?

## Methods

**Fig. 1:**
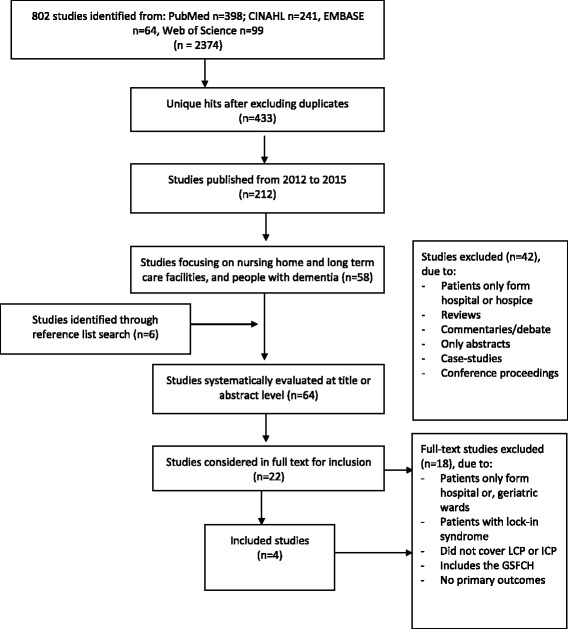
PRISMA based flowchart of the systematic search and review process

Following the PICO (patient, problem or population, intervention, comparison, control or comparator, and outcomes) method [14] (Table 1), we conducted a systematic literature search in the databases PubMed, CINAHL, EMBASE, and Web of Science with a search strategy including MESH terms and free text relevant for the LCP use in NHs and in people with dementia and terms related to psychometric testing and method development (validity, reliability, or responsiveness) (search strategy available as Additional file 1). The authors reviewed relevant MESH-terms and free text in collaboration with a trained medical librarian (RKL). Our search yielded 333 hits and 176 after excluding duplicates (Fig. 1). The review includes methodological, clinical, intervention, quantitative and qualitative studies that investigates the implementation (including staff education), and use of LCP. We also included two studies using GSFCH and LCP, even though the framework provides a broader approach, including ACP, over longer periods of time (i.e., not initiated at the end of life). In addition, we included two papers aimed to highlight LCP use in general or acute geriatric wards to perhaps find indications or recommendations regarding the assessment and treatment of pain and distressing symptoms in dying people with different stages of dementia or diagnoses of dementia. Since the LCP was developed in 1997 by Ellershaw et al., and later announced in 2001 [1], our search covered the whole time period between 1997 and august 2016. Based on the exclusion criteria (listed in Table 1), all authors screened potential manuscripts at abstract level (*n* = 176), and engaged in group discussions regarding manuscripts read in full text (*n* = 59) and borderline exclusion cases (see flow chart over exclusion process, Fig. 1).

**Table 1:** PICO-model indicating the inclusion and exclusion criteria of this study

Population	NH patients and their relatives.
Intervention	Liver Pool Care Pathway
Comparison	All studies using standard care group comparison, before/after comparison, as well as studies without standard means of comparisons were included.
Outcome	All outcomes both qualitative and quantitative were included.
Exclusion criteria	Studies only including home-dwelling and hospital patients
	Studies only including specific diagnoses (e.g., heart failure, cancer)
	Studies only using chart based interventions where patients/relatives are left on their own (e.g., advance directives without conversations).
	Studies that only focused on treatment limits (e.g., DNR, DNH). Publications such as case studies, chronicles, guidelines, protocols, unsystematic reviews and legal documents.
	Publications in in other languages than English and Scandinavian.
	Publications without abstracts.

The reference lists of included studies were reviewed, yielding six more manuscripts. In order to extract and synthesize the content of the included studies, manuscripts were read and discussed in group. We agreed on the content to be extracted, which were then organized in a data-extraction table. The table were piloted and discussed. For each included study, we extracted the following information: full manuscript reference, number of participants, study design and method, type of intervention and control condition (if applicable), time to follow-up, study setting, and outcomes. After agreeing on the format of data extraction, at least two of the co-authors read through each text independently and then verified the data-extraction in a discussion. Any unclear material was raised in group discussions. The authors agreed upon the grading of evidence according to an adapted Oxford Centre for Evidence-based Medicine – Levels of Evidence (available as Additional file 2) [15]. This system allows authors to grade studies on the basis of quality factors such as sample homogeneity, follow-up, definition of comparison group, and use of validated and relevant tests. The ten different levels of evidence defined in the system are available as Additional file 2. The review was designed in order to meet the systematic review standards of the PRISMA guidelines, and it was registered at the PROSPERO (registration number 42016045802) database.

## Results

Based on our systematic search we found that the LCP has generated a considerable number of publications and reports, including reviews and ethical and theoretical discussions. This production is still ongoing, with the latest contribution by Perkins E et al., [13]; a report on the end of life care in intensive care units and NHs. It is noteworthy that few publications are peer-reviewed original research. In total, we identified 12 studies investigating LCP use in NH patients and people with dementia. This also included one method paper which describes the development of a complex intervention [16]. The results are organized according to themes evident in the text and Tables 2, 3 and 4: i) Studies investigating the use of LCP in NHs and people with dementia, ii) Studies investigating the use of the GSFCH and LCP in NHs and people with dementia, and iii) Studies investigating the effect or further development of LCP in acute or general geriatric ward.

**Table 2:** Summary of the eight original studies investigating the effect of using the Liverpool Care Pathway (LCP) in nursing homes (NH)

1st author, year, nationality, Grading	Design/participants	Study objective	Outcome measures	Results
Watson J, 2006 [12], UK	Mixed method: qualitative and quantitative data were collected in 8 NH before, during and after the implementation of the LCP related to a 5-year action research project (Bridges Initiative) to develop practice around high quality end-of-life care in NHs	Explore barriers during implementing an integrated care pathway for the last days of life in nursing homes	- Documentary analysis of notes - Group interviews with trained staff, care assistants, GPs, relatives - Field notes - Participant observation	Six barriers through lack of: 1. palliative care knowledge, drugs and symptom control preparation for imminent death 2. knowing the dying process multidisciplinary team in NH 3. confidence in communicating readiness/ability to change
Grading: 2b				
Veerbeek L, 2008a [17, 18], The Netherlands	Non-controlled, pre- and post-intervention design in hospitals, NHs, at home: In the baseline period (11/2003–02/2005) 219 nurses participated for 220 deceased patients (*n* = 102 from NHs). In LCP intervention period (02/2005–02/2006) 253 nurses for 255 deceased patients (*n* = 114 from NHs).	Investigate effect of LCP on documentation of care, symptom burden and communication	Gender, age diagnoses After death of the patient, a nurse filled in the EORTC QlQ-C30 and VOICES, within 1 week after death. Relatives received the questionnaires 3 months after death.	LCP was used for 197 of the 255 dying patients (77%). Compared to baseline, the intervention had better documentation of care and lower symptom burden. The LCP implementation and use, pain and symptoms in NHs and persons with dementia were not reported, specifically. Study was not blinded.
Grading: 2b				
Veerbeek L, 2008b [17, 18], The Netherlands	Non-controlled, pre- and post-intervention design in hospitals, NHs, at home: In the baseline period (11/2003–02/2005) relatives participated for 56 NH patients. In LCP intervention period (02/2005–02/2006) relatives participated for 58 NH patients.	Investigate whether use of LCP affects relatives’ retrospective (4 months after death) evaluation of communication and level of bereavement	Views of Informal Carers—Evaluation of Services question-naire (VOICES)	Communication and end-of-life care were equally positively evaluated in both periods in the NH. In LCP group more relatives found information comprehensible, but difference was no longer significant after adjusting for differences in patient and relative characteristics.
Grading: 2b				
Van der Heide A, 2010 [19], The Netherlands	Non-controlled, pre- and post-intervention design in hospitals, NHs, at home. Patients with cancer; 83 patients with cancer died in the NHs. Data collection from physicians 1 week after death; from relatives 2 month after death	Retrospective evaluation of end-of-life decision-making practices for cancer patients who died in each of these settings and assessed the impact of LCP	C30 for physicians and relatives	80 physicians and 51 relatives filled in C30. Patients were included regardless the use of LCP (*n* = 40 used LCP in the NH). LCP had no significant impact on general drugs use during the last 3 days of life. Physicians estimated that drugs shortened life in 33% of NH patients. Parenteral sedation was used in 33% of NH patients. LCP reduced life-shortening drugs but it is not clear in what type of setting.
Grading: 3b				
Clark JB, 2012 [20], New Zealand	Mixed method: questionnaire sent to 194 health personnel from three NHs, 12–18 months after implementing LCP. 26 respondents. Qualitative interviews, both one-on-one (w/ one nurse, three physicians, one manager) and focus group (15 participants).	Investigate health personnel’s experience of LCP used in dying patients	- non-validated questionnaire (10 sider, 55 questions) - interviews	26 responders (13% response rate); 12/55 were reported: LCP-use positively evaluated in terms of communication, documentation, symptoms management, and education. The implementation of LCP was not described.
Grading: 4				
Lokker ME, 2012 [21], The Netherlands	Retrospective survey (2 months after death) including relatives/health personnel of persons who died in a hospital (117), NH (67), own home (82). Persons with reduced cognitive capacity were excluded, 70% cancer. LCP implemented midway through the study period (11/2003–02/2006)	Investigate if LCP use has an effect on how well the patients understand their terminal condition and dying as imminent.	28 symptoms from the EORTC QLQ-C30	LCP used in 33% of participants. The comprehension of dying as imminent was not related to LCP, age or diagnosis. LCP use, pain and symptoms in NHs and persons with dementia were not reported. Implementation of LCP was not described.
Grading: 2b				
Brannstrom M, 2015 [22], Sweden	Retrospective controlled survey (1 month), including relatives/health personnel of persons who died in 19 NHs (intervention *n* = 71, control *n* = 64) in one Swedish municipality (06/2009–10/2011). 3-h LCP education.	Investigate the effect of LCP on pain, symptoms and QoL in the end of life, before/after implementation	-ESAS -VICES	Dyspnea and nausea was better treated in the LCP treated group (evaluated by VICES and ESAS respectively). Other symptoms were not mentioned.
Grading: 2b				
Raijmakers N, 2015 [23], The Netherlands	Qualitative study including LCP managers from 10 organizations (four hospices/palliative NH units, three hospitals and three home care services	Identify barriers and promotors for the implementation of LCP	Telephone interviews and focus groups	Barriers/promotors for implementation of LCP in NHs/persons with dementia were not specified in results/discussion.
Grading: 4				

C30 = Cancer Quality of Questionnaire (for relatives and physicians); *DNAR* Do Not Attempt Resuscitation, *ESAS* Edmonton Symptom Assessment System, PCU = Palliative Care Unit, *VOICES* Views of Informal Carers - Evaluation of Services; EORTC QLQ-C30 = A core quality of life questionnaire covering general aspects of health-related quality of life and disease- or treatment-specific questionnaire modules

**Table 3:** Summary of the 2 original studies investigating the effect of using the GSFCH = Gold Standards Framework for Care Homes and Liverpool Care Pathway (LCP) in nursing homes (NH). DNAR = Do Not Attempt Resuscitation

1st author, year, nationality	Design/participants	Study objective	Outcome measures	Results
Hockely J, 2010, UK [24]	Qualitative interviews of bereaved relatives, pre−/post-implementation of the GSFCH and LCP in 7 Scotch NHs. Notes of 228 patients who had died prior to and during the project were examined, alongside a staff audit looking at the effect of GSFCH and LCP.	Investigate the implementation strategy of high facilitation including NH visits every 10–14 days and in-house staff training over 18-month.	In-depth evaluation of professional practices and residents outcomes	High staff turn-over (>33%). Use of LCP rose from 3% to 30%. Three of 7 NHs used it regularly. General increase of DNAR and ACP and reduction of hospital admissions/deaths. Pain, symptoms, medication use not reported. Isolated LCP effect unclear.
Grading: 2b				
Watson J, 2010 [25], UK	Qualitative interviews with 22 bereaved relatives/friends before (08/06–01/07) and 14 bereaved relatives/friends and six care home managers after (01/08–04/08) implementation of the GSFCH and LCP into 7 Scotch NHs.	Evaluate the impact on the quality of end-of-life care of the GSFCH and LCP. Implementation reported elsewhere (Hockely et al. 2010)	Content analyses of the 7Cs of the GSFCH related to GSFCH implementation	“Some NHs were using the LCP”. One relative comments that instructions were followed academic such as a textbook. Meanwhile all patients are individually. Another relative recognized that the patient was “changed every single day”. Unclear how many people were treated with LCP of NHs which used the LCP.
Grading: 4

**Table 4:** Summary of the 2 original studies investigating the effect/further development of Liverpool Care Pathway (LCP) in AGW = Acute Geriatric Ward; GGW = General Geriatric Ward; GSFCH = Gold Standards Framework for Care Homes; PCU = Palliative Care Unit

1st author, year, nationality	Design/participants	Study objective	Outcome measures	Results
Ekestrom ML, 2014, Sweden [26]	Non-controlled, before - after implementation of the LCP in a PCU and in a GGW. 44 family members of diseased patients from GGW participated (21 before and 23 after LCP implementation)	Explore family members’ experiences of end-of-life care	Questionnaire 3–6 months after death Comparisons between the samples by non-parametric tests	Physicians’ ability to listen to family members’ concerns suggested increasing. Very small numbers in sub-groups and not-randomized study design make interpretability of results questionable.
Grading: 2b				
Verhofstede R, 2015 [16], Belgium	Phase 0–1 methodology study based on Medical Research Council framework to develop and evaluate a complex intervention Phase 0 consists of a review of existing LCP programs from UK, Italy, and the Netherlands (NL), to identify factors for a successful LCP implementation and analysis of the concerns raised in the UK. Phase 1, is the development a care program for last days of life for older patients dying in AGW based on phase 0.	Develop a new care program to improve care in the last days of life for older patients dying in AGW	- Review of LCP-programs developed in UK and used in Italy and Netherland - Development of a care program	Pre-clinical phase: Non-systematic review identified three common documents: LCP documents, supportive documents, implementation guide. Modelling phase: Development of Care Guide for last days of life: supportive documentation, implementation guide for older hospital population. Dementia or ACP are not mentioned as an outstanding challenge in evaluation and treatment of pain and symptoms or communication
Grading: 2b				

### Studies investigating the use of LCP in nursing homes and people with dementia

In a study from Scotland, Watson et al., [12] conducted a mixed method study to investigate the barriers of the implementation of the Liverpool care pathway for the last days of life in nursing homes. Qualitative and quantitative data were collected in eight nursing homes before, during and after the implementation of the LCP, following a 5-year action research project (Bridges Initiative) which aimed to enhance the quality of end-of-life care in NHs. Through analyses of field documentation, group interviews with trained staff, care assistants, GPs, and relatives, six barriers of the implementation of LCP in this type of caring settings were identified. Firstly, the authors highlighted lack of knowledge regarding palliative care, drug use and symptom control in the NH as a key barrier. Further, staff may not be able to recognize when dying is imminent and thus the necessary preparation would not be possible. The third barrier was lack of understanding the dying process, possibly because NHs has been under-resourced for a long time. Additional barriers were lack of a multidisciplinary team with shared decision making, training in communication, and readiness and ability to change. No patient outcomes were reported.

A study from The Netherlands (NL), contributed to three papers by Veerbeek et al., [17, 18], and van der Heide et al., [19]. The trial used a pre- and post-intervention design of LCP and included a university and a general hospital, two NHs, and a home care organization. The majority of the patients had cancer; LCP was used for 197 of the 255 dying patients (77%). Compared to baseline, the intervention had better documentation of care and lower symptom burden [17]. The second [18] and third article [19] evaluated the effect of LCP on communication and level of bereavement, and on end-of-life decision-making practices, respectively. Compared to baseline, no differences were found in communication and end-of-life care in NHs. However, in these publications the implementation and use of the LCP in participating NHs and people with dementia specifically are not reported, neither were patient outcomes.

In a study from New Zealand, Clark et al., [20] used a mixed method study design to describe the experience of LCP use for dying patients among health professionals in the setting of three aged residential care facilities. In this study, a questionnaire, uniquely developed for the study (10 pages, 55 questions), was sent to 194 employees 12–18 months after LCP distribution. Nine nurses, two working on a temporary contract, ten nursing assistants, three physicians and, two unspecified workers returned the questionnaire (*N* = 26, response rate = 13%). Due to a high missing-rate, the results were based on a selection of 12 items from the 55-question survey. In addition, focus group interviews with 15 participants and 5 one-on-one telephone calls were completed. The participants provided positive ratings on LCP in general. However, LCP implementation strategies and the questionnaire’s validity and reliability were not reported; no clinical outcome measures were used in this study.

In a retrospective Dutch survey including relatives and healthcare professionals by Lokker et al., [21], questionnaires were distributed 2 months after a patient’s death. The study included cases where the patients died in hospitals (*n* = 117), NHs (*n* = 67) or in their own homes (*n* = 82). The questionnaire investigated whether the use of LCP had an effect on the patient’s understanding of death as imminent. LCP was introduced halfway through the study period (11/2003 to 02/2006) and was used in approximately one-third of the cases. Implementation strategies were not described. While the study reported that 70% of the patients had cancer, the prevalence of dementia was not disclosed. The study concluded that the use of LCP had no effect on the patient’s understanding of their situation in the dying process.

The before-after controlled retrospective study by Brannstrom et al., [22], included 19 residential care homes with 837 patients in one Swedish municipality. About half of the study participants had dementia [22]. The LCP implementation strategy consisted of a three-hour educational session for the staff, and an extended training of senior nurses, responsible for the staff. This survey-based study included relatives and nursing staff *1 month after a patient’s death*, to evaluate the effect of LCP on pain, distressing symptoms, and QoL. LCP was introduced halfway through the study period (06/2009 to 10/2011). Relatives of 71 of 220 patients in the intervention group (LCP) and 64 of 204 in the control group answered to the questions. The outcome measures were the Edmonton Symptom Assessment System (ESAS) and VOICES. The study found that patients in the intervention group were better treated for nausea and dyspnoea assessed by ESAS, and dyspnoea assessed by VOICES. They found no effect on QoL or symptoms such as pain and anxiety.

Finally, the study by Rajmakers et al., [23] was qualitative, involving LCP-implementation managers (stakeholders) from 10 Dutch organizations (three hospitals, four hospices or palliative departments of NHs, and three home services). The study used telephone interviews and focus group interviews with the stakeholder (consultants, network coordinators, and project leaders) to identify barriers and promoters for the LCP implementation. In this study, participation of one NH is mentioned but it is unclear whether NHs staff or stakeholders were included in the study interview. No patient outcomes were addressed.

### Studies investigating the use of the GSFCH and LCP in nursing homes and people with dementia

Another larger study from Scotland was carried out to implement and evaluate the implementation of the GSFCH and LCP in seven nursing homes in one Community Health partnership. The implementation period lasted for 18 months and was undertaken by an experienced palliative care nurse. In the first paper, Hockely et al., [24] described in-depth evaluation of professional practices and residents` outcomes. During the study period, the use of LCP rose from 3% to 30% and three of seven NHs used it regularly. Researchers also found a general increase in Do Not Attempt Resuscitation (DNAR) orders and ACP, and reduction of hospital admissions or hospital deaths. However, the assessment and treatment of pain and distressing symptoms in the dying old or medication use are not reported. The GSFCH has a much broader approach to end-of-life care, unfortunately, the estimated isolated LCP effect was not reported. The other paper by Watson et al., [25], reported the implementation process of the GSFCH and LCP by qualitative interviews. Watson et al. [12] described the barriers of LCP implementation and use in NH settings such as, the need for palliative care knowledge, proper drugs and symptom control, the preparation for imminent death, knowing the dying process, multidisciplinary teamwork, confidence in communicating, and readiness to change. Noticeably, the authors point out that the intervention took time to implement, and that restricted time to follow-up resulted in interviews being completed before LCP was fully implemented.

### Studies investigating the effect or further development of LCP in acute or general geriatric ward

To give a broad impression of the LCP-use in people with dementia, we also searched the literature for the key word “geriatric*” to include those who are in need for admission to acute geriatric ward or general geriatric ward. A before and after LCP implementation study in a palliative care unit and in a general geriatric ward was described by Ekestrom et al., [26] and included 44 family members of deceased patients (21 before and 23 after LCP implementation). Compared to control, relatives in the intervention group suggested that physicians’ ability to listen to family members’ concerns was increased. There were no differences between the groups in relation to pain and symptom management. However, small numbers of participants in the LCP sub-groups and a not-randomized (before-after) study design make generalization of results challenging.

The second study, by Verhofstede et al., [16] took a methodological approach, which was described as two phase study: Phase 0 (preclinical phase) consisted of a non-systematic review to evaluate factors for successful LCP implementation in the UK, Italy, and NL. Based on the results, phase 1 (modelling phase) developed a Care Guide for the Last Days of Life, supportive documentation, and an implementation guide addressing the older acute geriatric hospital population. People with dementia were not mentioned in this context. This is the only method paper describing the development of the LCP systematically. However, the study has not yet included patients; the focus relies on the hospital setting and excludes people with dementia.

### Results summarized in accordance to the research questions

In summary, regarding the research question 1*,* we found no studies that described LCP’s measurement characteristics pertaining to validity, reliability, and responsiveness. Moreover, we could not identify studies on the adaption of the LCP to become appropriate for NHs and among people with dementia.

Relevant to research question 2, our systematic search identified two before–after intervention studies [17–19, 26], two retrospective surveys [21, 22], two qualitative studies [23–25], two mixed-method studies [12, 20], and one methodology study [16]. There were no randomized controlled trials (RCT) or prospective studies on LCP use in NHs or people with dementia. None of these studies had a blinded design.

With research question 3 we aimed to highlight implementation strategies to promote and facilitate LCP in clinical NH settings. Most included papers highlight the necessity of proper implementation. However, only the study by Brannstrom et al., investigated the effect of LCP on pain, symptoms, and QoL in the end of life, before and after LCP implementation [22]. Yet, this was a retrospective investigation. Meanwhile, we found no studies describing strategies for discontinuation of medications, procedures for nutrition and hydration, or testing of clinical recommendations in NHs. People with different stages or types of dementia were not mentioned in any publication.

## Discussion

This systematic review investigated the use of LCP in NHs and to what extent it has been adapted from its original use in cancer patients to the use among multimorbid NH patients and people with dementia. We also included papers describing the combined use of GSFCH and LCP, and the use of LCP in acute geriatric wards and general geriatric wards.

In general, the evidence for methodological LCP adaptions for NHs and people with dementia, and the implementation strategies and use of LCP in these populations is weak, almost absent, and existing results are not definitive. We acknowledge that RCTs are difficult to complete in this population, and believe, that other designs could also provide important evidence. However, our main concern is that we did not find studies that document the development and testing of the instrument by including elderly multimorbid patients or people with dementia. This suggests that LCP is not adapted and measures of validity, reliability or responsiveness are lacking in this setting. Research investigating an instrument’s psychometric properties is a prerequisite, and should include testing of various aspects of validity, reliability (intra−/interrater, retest etc.) and responsiveness (identifying change after treatment) [32]. NH patients are fragile, multimorbid, and dependent, with over 80% affected by dementia [27]. To estimate imminent dying, and the assessment and treatment of pain and distressing symptoms is challenging in this population [28–30].

We found 12 publications based on nine clinical studies of varying research quality. Only the study by Brannstrom et al. was controlled [22]. Meanwhile, the study had low internal validity because LCP implementation was completed halfway through the study period. The study was not blinded, which can contribute to measurement errors and Hawthorne effect [31]. Other studies had low response rates with potential errors and biases, unclear inclusion procedures of people with dementia, or lack of prevalence for LCP use [20, 21]. Documentation of development and testing of a method in a clinically relevant setting is a prerequisite before it may be implemented as a clinical standard [32]. Conclusive recommendations based on aggregated evidence are nearly impossible to make.

Despite the scientific weaknesses there are also some positive actions worth to be mentioned. The Scottish study by Hockley and Watson reports qualitative analyses in order to the use of GSFCH and LCP in seven NHs [24, 25], although the isolated implementation and effect of LCP was not reported. The results demonstrate the necessity of a broader communication process, starting early. Also, the article by Watson et al. [12] deserves attention because it describes the bottlenecks of LCP implementation and use in these settings and addresses potential problems in adopting it in NHs and to persons with dementia 2006. A more recent article highlights the further development of the LCP to meet the patients’ needs when they are at the end-of-life admitted to an acute geriatric ward or general geriatric ward [16, 26]. This contribution outlines a stepwise, review process and plans to adapt LCP for care for the elderly. Although this approach does not mention different stages and types of dementia or the necessity of ACP, the work demonstrates the complexity of clinical field that LCP originally was set to resolve.

The present review confirms findings from previously published review articles [33–38]. An updated version (09/2010 to 06/2013) [34] of a preceding Cochrane report (1950 to 09/2009) [33] described the effect of LCP on symptoms relief at the end-of-life when compared with usual care in hospitals, NHs, and at home. The report concluded that there are no controlled studies of good quality examining the efficacy of LCP on symptom intensity as well as their quality of life and dying. Potential barriers such as unpredictable end-of-life trajectories in non-cancer patients, and lack of skilled care providers were also discussed [38]. In general, due to the insufficient validated outcome measures and use of control conditions in clinical studies, these reviews did not draw any conclusions based on existing literature and even recommended that LCP use should be avoided for use in NH settings and among people with dementia, until such studies exist [38].

**Fig. 2:**
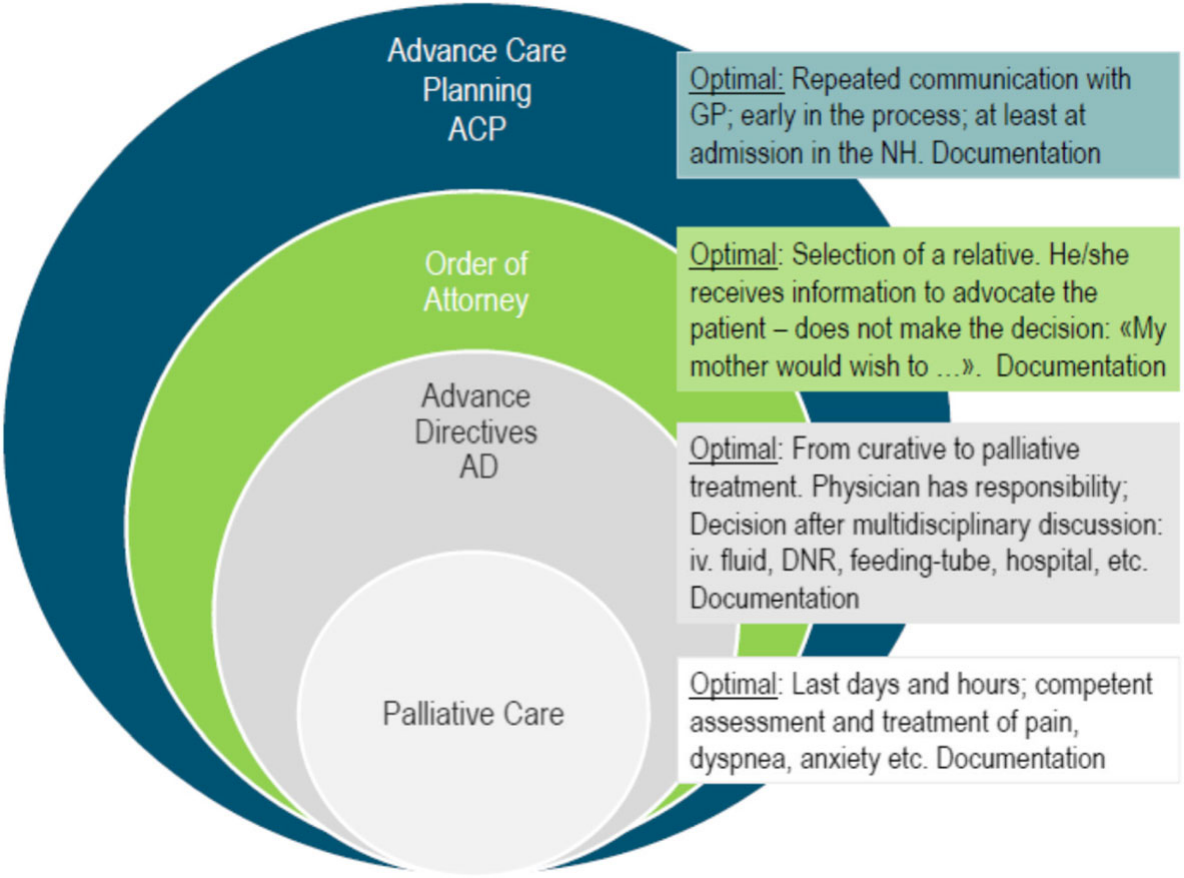
Levels of care and communication to prepare for later stages of life

A good death is significant for dying patients and their families and the complex should start early in the patient’s individual disease history (Fig. 2). In a commentary published in *The Lancet*, Currow and colleagues call attention to the detail that LCP was used without adequate patient assessment by an experienced clinician and that the implementation was flawed [39]. The authors question how UK officials could launch the LCP without research-based support. Economic interests were mentioned as an explanation and health economic reports oppose further financial support for LCP-implementation [40]. It is further not acceptable to simply transfer the results from cancer patients to other patient groups with or without dementia [41, 42]. Among patients with dementia, it is far more difficult to predict the end-of-life than in cancer patients [43]. The article, “Less ticking the boxes, more providing support” by Di Leo et al., [44] highlights health professionals’ concerns related to LCP use, such as problems with organizing participation in education and training programs; difficulties in including physicians; identification of the patient as dying; and interpretation of observations presented in the LCP tick-off form and documentation. Although there is existing evidence-based knowledge about pain assessment and treatment of people with dementia [45, 46], there is currently only one prospective study that investigates pain and symptom treatment in dying NH patients, either with or without dementia [47]. Almost 40% died unexpectedly or were not recognised as being dying and most symptoms, including pain (46%) and dyspnea (53%), were still frequent at day of death. Findings are of clinical importance because typical behaviour of pain may resemble behaviours that are common in dementia diseases [45, 46]. This can lead to incorrect interpretation and treatment of symptoms. In a NH, the patient’s primary contact is often a nursing assistant with varied training in end-of-life care. It is an unfair task to ask an unqualified health worker to tick-off the Yes or No box to determine whether a dying person with dementia has pain, dyspnoea or nausea.

Highlighted by the Neuberger Report, the LCP, *when used in the right hands*, can provide a model of good practice for the last days and hours of life [11]. However, *in the wrong hands*, the report concluded that the LCP has been used as an excuse for poor quality of care. Summarized by Wrigley A., [48] the concerns are largely based on misconceptions about or improper implementation of the LCP and a misunderstanding what the LCP is designed to do. He also argues that the complete retraction of LCP, could be compared to retracting morphine or insulin from the marked, “because its correct use is beneficial but some people incorrectly use it”. Meanwhile the premise for using morphine or insulin is high competence, high quality diagnostics, and the presence of physicians. With this we argue, that a precautionary stance is pertinent in this context. Subsequently, an intense effort was initiated in the UK to ensure competent treatment in the terminal stage. The recently published National Clinical Guidelines (NICE) [49], *Care of dying adults in the last days of life*, presents diagnostic and treatment alternatives and thereby supports other international initiatives [9, 10, 43, 50]. Meanwhile, recommendations highlighted in NICE are based on mainly randomised clinical studies which were conducted in hospitals or home care services by inclusion of cancer patients. NH patients and people with dementia are not considered. In Norway, almost 50% of the dying population dies in a NH; 80% have dementia. Even though financial gain from using the LCP are not overtly expressed as a goal, proper and regular education and training of all staff, would be more costly and time-consuming than the use of the LCP alone. After criticism in social media in Norway (Høeg, Morgenbladet 2015), the title of the LCP and some segments are rephrased. However, as outlined by O’Dowd [51], a name change is not good enough. Meanwhile, staff competence as prerequisite to use this pathway is entirely unknown.

If LCP is not the optimal intervention in NHs, what should replace it, and how would this be better? In a comprehensive white paper on behalf of the European Association for Palliative Care, by Van der Steen and colleagues, a Delphi expert rating evaluated 11 domains and 57 recommendations on palliative care and organized the following domains as important for end of life care in people with dementia: 1. Optimal treatment of symptoms and providing comfort, 2. Person-centred care, communication and shared decision-making, 3. Family care and involvement, 4. Societal and ethical issues, 5. Avoiding overly aggressive, burdensome or futile treatment, 6. Education of the health care team, 7. Psychosocial and spiritual support, 8. Continuity of care, 9. Setting care goals and advance planning, 10. Applicability of palliative care, 11. Prognostication and timely recognition of dying. Similar topics were identified for research priorities. Most of all, this process demonstrates that end of life care in dementia, is not only isolated to the last hours of life, it is a long preparing process involving complex and multimorbid patients lacking ability to provide informed consent, and their families. These challenges need to be met by building competence and changing attitudes in NH over time.

## Conclusions

Our systematic review demonstrates that the LCP has not been adapted to the individual needs of dying nursing home patients and people with dementia. In particular, the validity, reliability, and responsiveness of the tool have not been tested in the clinical setting. As such, the LCP is not an evidence-based procedure and health care authorities have to judge carefully whether the recommendation of the LCP is justifiable. After changing the name of the procedure, the LCP is still in use in many countries, as a low-cost camouflage of the real need for education and competence in nursing homes.

## Additional files

Additional file 1: Full search strategy – list of all terms used to search MEDLINE, EMBASE, CINAHL and WEB Sci. (DOCX 19 kb)

Additional file 2: The Quality grading system – describes the Oxford Centre for Evidence-based Medicine – Levels of Evidence system used in the present review. (DOCX 17 kb)

## Abbreviations

ACP: Advance care planning
ESAS: Edmonton symptom assessment system
GSFCH: Gold standards framework for care homes
LCP: Liverpool care pathway
NICE: National Institute for Health and Care Excellence
PICO: Patient, problem or population, intervention, comparison, control or comparator, and outcomes
QoL: Quality of life
VOICES: Views of informal carers – evaluation of services
