# Erratum to: The Liverpool Care Pathway: discarded in cancer patients but good enough for dying nursing home patients? A systematic review

**DOI:** 10.1186/s12910-017-0211-z

**Published:** 2017-08-25

**Authors:** Bettina S. Husebo, Elisabeth Flo, Knut Engedal

**Affiliations:** 1 Centre for Elderly and Nursing Home Medicine, Department of Global Public Health and Primary Care, University of Bergen, Bergen, Norway; 2 Bergen Municipality, Bergen, Norway; 3 Department of Clinical Psychology, University of Bergen, Bergen, Norway; 4 Norwegian National Advisory Unit on Ageing and Health (Ageing and Health), Vestfold hospital and Oslo universitet hospital, Ullevaal, Oslo, Norway

## Erratum

After publication of the article [1], it has been brought to our attention that the wrong title was used on initial publication. The correct title of this article is “The Liverpool Care Pathway: discarded in cancer patients but good enough for dying nursing home patients? A systematic review”. The original version of the article has been updated to reflect this.
