# Supplementary material for: The Liverpool Care Pathway: a systematic review discarded in cancer patients but good enough for dying nursing home patients?
Source: BMC Med Ethics. 2017 Aug 9;18:48. doi: 10.1186/s12910-017-0205-x (PMC5551006; doi:10.1186/s12910-017-0205-x)
Supplement: Supplementary file 1 — Full search strategy – list of all terms used to search MEDLINE, EMBASE, CINAHL and WEB Sci. (DOCX 19 kb) [file 12910_2017_205_MOESM1_ESM.docx]

**Search strategies**
**Database: Epub Ahead of Print, In-Process & Other Non-Indexed Citations, Ovid MEDLINE(R) Daily and Ovid MEDLINE(R) <1946 to Present>, 2. Sept. 2016**1 Terminally Ill/ (5878)
2 exp Terminal Care/ (44547)
3 Palliative Care/ (45366)
4 (terminally ill or terminal care or end-of-life care or palliative care or dying or end-stage or hospice care).ti,ab,kw. (107153)
5 1 or 2 or 3 or 4 (164223)
6 Critical Pathways/ (5427)
7 (liverpool care pathway or LCP or clinical pathway or critical pathway or integrated pathway or end-of-life care pathway).ti,ab,kw. (3734)
8 6 or 7 (8089)
9 5 and 8 (421)
10 homes for the aged/ or exp nursing homes/ (39092)
11 Hospices/ (4600)
12 Geriatrics/ (28236)
13 exp Primary Health Care/ (118481)
14 (nursing home* or "home* for the aged" or hospice* or geriatric* or primary health care).ti,ab,kw. (106804)
15 10 or 11 or 12 or 13 or 14 (239655)
16 9 and 15 (99) after removing duplicates 89; after hand-removing duplicates **78 articles in MEDLINE**

**Database: Embase <1974 to 2016 September 01>,** 2. Sept. 2016
1 exp terminally ill patient/ (7168)
2 exp terminal care/ (53533)
3 palliative therapy/ (62665)
4 (terminally ill or terminal care or end-of-life care or palliative care or dying or end-stage or hospice care).ti,ab,kw. (146007)
5 1 or 2 or 3 or 4 (213362)
6 clinical pathway/ (7160)
7 (liverpool care pathway or LCP or clinical pathway or critical pathway or integrated pathway or end-of-life care pathway).ti,ab,kw. (5129)
8 6 or 7 (10392)
9 5 and 8 (538)
10 health care facility/ or hospice/ or nursing home/ (113025)
11 home for the aged/ (11251)
12 exp geriatrics/ (47671)
13 exp primary health care/ (128452)
14 (nursing home* or "home* for the aged" or hospice* or geriatric* or primary health care).ti,ab,kw. (126406)
15 10 or 11 or 12 or 13 or 14 (349176)
16 9 and 15 (138) after removing duplicates 72; after hand-removing duplicates **59 articles in EMBASE
CINAHL (Ebsco), 2. Sept. 2016**S1 (MH "Terminally Ill Patients+") 8,220
S2 (MH "Terminal Care+") 40,253
S3 TI ( "terminally ill" or "terminal care" or "end-of-life care" or "palliative care" or dying or end-stage or "hospice care" ) OR AB ( "terminally ill" or "terminal care" or "end-of-life care" or "palliative care" or dying or end-stage or "hospice care" ) 32,722
S4 S1 OR S2 OR S3 56,104
S5 (MH "Critical Path") 3,391
S6 TI ( "liverpool care pathway" or LCP or "clinical pathway" or "critical pathway" or "integrated pathway" or "end-of-life care pathway" ) OR AB ( "liverpool care pathway" or LCP or "clinical pathway" or "critical pathway" or "integrated pathway" or "end-of-life care pathway" ) 972
S7 S5 OR S6 3,787
S8 S4 AND S7 380
S9 (MH "Nursing Homes+") OR (MH "Nursing Home Patients") 24,173
S10 (MH "Hospices") OR (MH "Hospice Patients") 2,547
S11 (MH "Geriatrics") 2,808
S12 (MH "Primary Health Care") 33,360
S13 TI ( nursing home* or "home* for the aged" or hospice* or geriatric* or "primary health care" ) OR AB ( nursing home* or "home* for the aged" or hospice* or geriatric* or "primary health care" ) 40,577
S14 S9 OR S10 OR S11 OR S12 OR S13 86,459
S15 S8 AND S14 63, after removing duplicates 37; after hand-removing duplicates **31 articles in CINAHL**

**Web of Science, Indexes=SCI-EXPANDED, SSCI, A&HCI, ESCI Timespan=All years
 (Thomson & Reuters), 2. Sept. 2016**# 4 33 #3 AND #2 AND #1 after removing duplicates 10; after hand-removing duplicates **8 articles in Web of Science**# 3 88,963 TOPIC: ("nursing home*" or "home* for the aged" or hospice* or geriatric* or "primary health care")
# 2 6,137 TOPIC: ("liverpool care pathway" or LCP or "clinical pathway" or "critical pathway" or "integrated pathway" or "end-of-life care pathway")
# 1 558,827 TOPIC: ("terminally ill" or "terminal care" or "end-of-life care" or "palliative care" or dying or end-stage or "hospice care")

**In total 333 articles; 126 duplicates were removed leaving 207 articles. After hand-removing further 31 duplicates were excluded, remaining in total 176 articles.**
