# Supplementary material for: The Liverpool Care Pathway: a systematic review discarded in cancer patients but good enough for dying nursing home patients?
Source: BMC Med Ethics. 2017 Aug 9;18:48. doi: 10.1186/s12910-017-0205-x (PMC5551006; doi:10.1186/s12910-017-0205-x)
Supplement: Supplementary file 2 — The Quality grading system – describes the Oxford Centre for Evidence-based Medicine – Levels of Evidence system used in the present review. (DOCX 17 kb) [file 12910_2017_205_MOESM2_ESM.docx]

Oxford Centre for Evidence-based Medicine – Levels of Evidence (March 2009) <http://www.cebm.net/oxford-centre-evidence-based-medicine-levels-evidence-march-2009/>

| **Level** | **Therapy / Prevention, Aetiology / Harm** | **Prognosis** | **Diagnosis** | **Differential diagnosis / symptom prevalence study** | **Economic and decision analyses** |
| --- | --- | --- | --- | --- | --- |
| 1a | SR (with homogeneity*) of RCTs | SR (with homogeneity*) of inception cohort studies; CDR”  validated in different populations | SR (with homogeneity*) of Level 1 diagnostic studies; CDR”  with 1b studies from different clinical centres | SR (with homogeneity*) of prospective cohort studies | SR (with homogeneity*) of Level 1 economic studies |
| 1b | Individual RCT (with narrow Confidence Interval”¡) | Individual inception cohort study with > 80% follow-up; CDR”  validated in a single population | Validating** cohort study with good” ” ”  reference standards; or CDR”  tested within one clinical centre | Prospective cohort study with good follow-up**** | Analysis based on clinically sensible costs or alternatives; systematic review(s) of the evidence; and including multi-way sensitivity analyses |
| 1c | All or none§ | All or none case-series | Absolute SpPins and SnNouts” “ | All or none case-series | Absolute better-value or worse-value analyses ” ” ” “ |
| 2a | SR (with homogeneity*) of cohort studies | SR (with homogeneity*) of either retrospective cohort studies or untreated control groups in RCTs | SR (with homogeneity*) of Level >2 diagnostic studies | SR (with homogeneity*) of 2b and better studies | SR (with homogeneity*) of Level >2 economic studies |
| 2b | Individual cohort study (including low quality RCT; e.g., <80% follow-up) | Retrospective cohort study or follow-up of untreated control patients in an RCT; Derivation of CDR”  or validated on split-sample§§§ only | Exploratory** cohort study with good” ” ”  reference standards; CDR”  after derivation, or validated only on split-sample§§§ or databases | Retrospective cohort study, or poor follow-up | Analysis based on clinically sensible costs or alternatives; limited review(s) of the evidence, or single studies; and including multi-way sensitivity analyses |
| 2c | “Outcomes” Research; Ecological studies | “Outcomes” Research |  | Ecological studies | Audit or outcomes research |
| 3a | SR (with homogeneity*) of case-control studies |  | SR (with homogeneity*) of 3b and better studies | SR (with homogeneity*) of 3b and better studies | SR (with homogeneity*) of 3b and better studies |
| 3b | Individual Case-Control Study |  | Non-consecutive study; or without consistently applied reference standards | Non-consecutive cohort study, or very limited population | Analysis based on limited alternatives or costs, poor quality estimates of data, but including sensitivity analyses incorporating clinically sensible variations. |
| 4 | Case-series (and poor quality cohort and case-control studies§§) | Case-series (and poor quality prognostic cohort studies***) | Case-control study, poor or non-independent reference standard | Case-series or superseded reference standards | Analysis with no sensitivity analysis |
| 5 | Expert opinion without explicit critical appraisal, or based on physiology, bench research or “first principles” | Expert opinion without explicit critical appraisal, or based on physiology, bench research or “first principles” | Expert opinion without explicit critical appraisal, or based on physiology, bench research or “first principles” | Expert opinion without explicit critical appraisal, or based on physiology, bench research or “first principles” | Expert opinion without explicit critical appraisal, or based on economic theory or “first principles” |
